# Doxycycline Attenuates Doxorubicin-Induced Cardiotoxicity by Improving Myocardial Energy Metabolism in Rats

**DOI:** 10.3390/jcdd9080254

**Published:** 2022-08-08

**Authors:** Danielle Dantas, Amanda Gomes Pereira, Anderson Seiji Soares Fujimori, Ana Paula Dantas Ribeiro, Carol Cristina Vágula de Almeida Silva, Marina Gaiato Monte, Camila Renata Corrêa, Ana Angélica Fernandes, Silmeia Garcia Zanati Bazan, Paula Schmidt Azevedo, Marcos Ferreira Minicucci, Sergio Alberto Rupp de Paiva, Leonardo Antônio Mamede Zornoff, Bertha Furlan Polegato

**Affiliations:** 1 Department of Internal Medicine, Botucatu Medical School, São Paulo State University (UNESP), Botucatu 18618687, Brazil; 2 Department of Pathology, Botucatu Medical School, São Paulo State University (UNESP), Botucatu 18618687, Brazil; 3 Department of Chemistry and Biochemistry, Institute of Biosciences, São Paulo State University (UNESP), Botucatu 18618687, Brazil

**Keywords:** doxycycline, myocardial energy metabolism, oxidative stress, MMP-2, collagen I, TIMP-4

## Abstract

Aim: Evaluate the influence of doxycycline, an anti-inflammatory and matrix metalloproteinase (MMP) inhibitor, on the attenuation of chronic doxorubicin-induced cardiotoxicity in rats. Methods: We allocated male Wistar rats into four groups: control (C), doxorubicin (D), doxycycline (inhibitor of MMP, IM), and Dox + doxycycline (DIM). Groups IM and DIM received doxycycline (5 mg/kg, IP) once a week for 4 weeks. In addition, 48 h after every doxycycline injection, groups D and DIM received Dox (5 mg/kg, IP). We performed echocardiogram and evaluated TIMP-4 and collagen I protein expression, MMP-2 activity, and oxidative stress and myocardial metabolism. Results: Doxorubicin promotes left atrium (LA) and left ventricle (LV) dilatation and decreases in LV fractional shortening, which was improved by doxycycline. Moreover, doxycycline attenuated the LV cardiomyocyte hypertrophy and collagen type I expression. Doxorubicin increased phosphofructokinase and decreased beta-hydroxyacyl Co-A dehydrogenase, pyruvate dehydrogenase, citrate synthase, and ATP synthase activity, which was partially attenuated by doxycycline. Lastly, doxycycline improved antioxidant enzyme activity in the DIM group. Conclusion: Doxorubicin increases oxidative stress and promotes changes in myocardial energy metabolism, accompanied by structural and functional changes. Doxycycline attenuated the doxorubicin-induced cardiotoxicity, at least in part, through changes in myocardial energy metabolism.

## 1. Introduction

Doxorubicin (Dox) is an effective antineoplastic agent used for the treatment of multiple cancer types, such as solid tumors and hematopoietic malignancies. Dox acts mainly as an inhibitor of the DNA topoisomerase II enzyme, found predominantly in proliferating cells, causing damage to the DNA and subsequently neoplastic cell death [1,2].

Despite the fact that Dox is considered a first-line treatment against cancer, its clinical use often causes cumulative and dose-dependent cardiotoxicity, occurring in up to 26% of the patients treated with the drug [3]. Once developed, Dox-induced cardiotoxicity carries a poor prognosis, and patients can have a high chance of eventually dying of heart failure instead of cancer itself [4,5]. Unfortunately, there is no effective method of preventing this complication, and no medication is highly effective in the treatment of cardiotoxicity.

Several underlying mechanisms have been proposed for this complication. The most accepted mechanism includes increased oxidative stress due to an excessive formation of reactive oxygen species (ROS). ROS generation in Dox-induced cardiotoxicity is related to changes in myocardial energy metabolism and mitochondrial dysfunction, impairment in iron handling, and the presence of nitric oxide synthase [6,7]. Moreover, Dox deactivates antioxidant substances such as glutathione peroxidase, superoxide dismutase, and catalase [1].

It is well-established that oxidative environments lead to the activation of matrix metalloproteinases (MMPs), a superfamily of zinc-dependent endopeptidases that primarily degrade extracellular matrix (ECM) proteins [8,9]. Specifically in the heart, MMP-2, also known as gelatinase, regulates extracellular matrix turnover and plays an important role in early myocardial injury, including in the acute model of Dox-induced cardiotoxicity [10,11,12]. The overactivation of MMP-2 in cardiac myocyte in response to increased oxidative stress creates an imbalance between matrix synthesis and the degradation of contractile proteins, which contributes to myocardium dysfunction and, ultimately, to progression to congestive heart failure [11,13]. MMP activity is regulated by several processes, such as glutathiolation, phosphorylation, and the specific inhibitor-denominated tissue inhibitor of matrix metalloproteinases (TIMP), the most important factor that determines MMP activity modulation. There are four types of TIMPs; the most abundant TIMP in the heart is TIMP-4 [14,15].

Doxycycline, a tetracycline antibiotic, has been studied extensively in several human and animal diseases due to its nonselective MMP inhibition [16,17,18,19]. Although the molecular mechanisms responsible for the inhibitory effect are not yet fully understood, doxycycline at a sub-antimicrobial dose may be an attractive pharmacological tool as a cardioprotective agent, considering that other MMPs may be involved in ventricular remodeling [16]. In fact, studies have suggested that early short-term doxycycline therapy ameliorates post-infarction remodeling and exerts protective effects on myocardial ischemia/reperfusion injury in experimental [20,21] and clinical settings [22].

Additionally, a recent study showed that doxycycline ameliorated Dox-induced cardiotoxicity by preventing myofilament lysis, titin proteolysis, and interstitial fibrosis in mouse hearts [23]. However, its inhibitor effect targeting specifically the myocardial matrix metalloproteinases has not yet been evaluated. 

Considering that doxycycline exerted antioxidant and anti-inflammatory effects in the treatment of chronic inflammation [18,24] and cardiovascular diseases [25,26,27], it is reasonable to postulate that doxycycline could act together by different pathways of myocardial aggression, such as improving energy metabolism [28]. Thus, we aimed to evaluate the influence of MMP inhibition by doxycycline on the attenuation of Dox-induced cardiotoxicity in rats. 

## 2. Material and Methods 

### 2.1. Study Design

This research protocol was approved by the Animal Ethics Committee of Botucatu Medical School (1259/2018) and complies with the Brazilian National Council for the Control of Animal Experimentation standards. In this study, 80 male Wistar rats, weighing 200–250 g each, were kept in a controlled environment with a 12 h light/dark cycle at 23 ± 2 °C and free access to regular food and water. We measured food intake and body weight during the experiment and randomly assigned the rats to 4 groups: control (C), Dox (D), doxycycline (inhibitor of MMP, IM), and Dox + doxycycline (DIM). The IM and DIM groups received an intraperitoneal (IP) injection of doxycycline (5 mg/kg) [29,30] once a week for 4 weeks, whereas groups C and D received an equivalent volume of IP sterile saline. Doxycycline is a potent non-selective MMP inhibitor, acting by interaction with the zinc and/or calcium atoms within the structural center of these enzymes, dislodging it from their receptors [18].

Additionally, 48 h after the doxycycline or saline injection, groups D and DIM received an IP injection of Dox (5 mg/kg) once a week for 4 weeks, and groups C and IM received an equivalent volume of IP sterile saline. Forty-eight hours after the last Dox or saline injection, we performed an echocardiogram on all the animals, followed by the isolated heart study (*n* = 10 per group) and euthanasia (thiopental injection, 120 mg/kg, IP). We then removed the hearts, washed them in fresh saline, and dissected, weighed, and stored them at −80 °C.

Eight animals from each group were prepared only for isolated heart study since retrograde perfusion used in this methodology may interfere in biochemical myocardial variables. The remaining animals were reserved for additional analysis. 

### 2.2. Echocardiogram

We anesthetized the rats with ketamine (50 mg/kg, IP) and xylazine (1 mg/kg, IP) and evaluated them by a transthoracic echocardiographic examination (General Electric Medical Systems, Vivid S6). We also evaluated the left ventricle diameter in systole (LVSD) and diastole (LVDD) and interventricular septum, left atrium, and aorta diameters. We analyzed systolic function via left ventricular fractional shortening ((LVDD—LVSD)/LVDD) and diastolic function via E and A waves, E/A ratio, and isovolumetric relaxation time. The examination was complemented by tissue Doppler evaluation of systolic (S’) and diastolic initial (E’) and late (A’) motion of the mitral ring in the lateral and septal wall. The S’ wave is related to systolic function, and the E’ and E’/E ratio are related to diastolic function [5,31].

### 2.3. Isolated Heart Study

After the echocardiography analysis, the rats received sodium thiopental (80 mg/kg, IP) and heparin (2000 UI, IP). We performed an isolated heart study using the Langendorff technique previously described in [11]. In short, we subjected the rats to sternotomy under artificial ventilation, and we isolated and cannulated the ascendant aorta; then, we started retrograding perfusion with a modified Krebs–Henseleit solution. The hearts were then transferred to the isolated heart study apparatus (Hugo Sachs Elektronik, March-Hugstetten, Germany), paced at 200 to 250 beats/min, and we inserted a latex balloon connected to a pressure transducer into the left ventricular cavity. The volume inside the balloon was increased to change the diastolic pressure from 0 to 25 mmHg. After each volume variation, we recorded diastolic and systolic left ventricular pressures, the maximum left ventricular pressure decrease rate (−dP/dt), and the maximum left ventricular pressure development rate (+dP/dt) [11].

### 2.4. Western Blot: TIMP-4 and Collagen I Protein Expression

We homogenized samples of the left ventricle (50 mg) in 500 µL of RIPA buffer and centrifuged them. We collected supernatants and quantified the total amount of protein by the Bradford method. We performed electrophoresis in acrylamide gels and transferred proteins to nitrocellulose membranes, which were incubated in 5% skimmed milk. Afterward, we incubated membranes with primary antibodies for TIMP-4 (rabbit polyclonal IgG, Boster Bio, Pleasanton, CA, USA) and type I collagen (mouse monoclonal IgG, Santa Cruz Biotechnology, Santa Cruz, CA, USA) for 12 h, followed by incubation with secondary antibodies. We performed immunodetection using chemiluminescence in an ImageQuant LAS camera imaging system (General Electrics, Uppsala, Sweden). We analyzed images via Gel-Pro 32 (Media Cybernetics, Rockville, MD USA) and used GAPDH (mouse monoclonal IgG, Santa Cruz Biotechnology, Santa Cruz, CA, USA) for normalization.

### 2.5. Zymography: MMP-2 Activity

MMP-2 was analyzed accordingly to previous studies [11,32]. In short, we diluted samples of left ventricular muscle (30 mg) in an extraction buffer (50 mM Tris pH 7.4, 0.2 M NaCl, 0.1% Triton X, and 10 mM CaCl_2_); then, we crushed and centrifuged them before quantifying the protein amount in the samples via the Bradford method. Then, we diluted samples with 20 µg of protein in a sample buffer (0.5 M Tris pH 6.8, 50% glycerol, and 0.05% bromophenol blue) and loaded them into wells of 8% SDS-polyacrylamide gel containing 1% gelatin. We performed electrophoresis at 110 V for 2 h in a Bio-Rad mini-protean system in the presence of the running buffer (Tris-Glycine-SDS pH 8.3). Afterward, we washed the gels with Triton X-100 2.5% and Tris-HCl 50 mM pH 8.4 and incubated them for 17 h at 37 °C with continuous agitation. Last, we stained the gel with 2.5% Coomassie brilliant blue followed by a 1 h bath in 30% methanol and 10% acetic acid at room temperature. We photographed the gels and analyzed them by Gel-Pro 3.2 (Media Cybernetics, Rockville, MD, USA). We included the same control sample in each gel to normalize the results. We confirmed the MMP-2 position in the gels by a recombinant rat/mouse MMP-2 standard (R&D System, Minneapolis, MN, USA).

### 2.6. Histology

After euthanasia, we cross-sectioned the left ventricle 5 mm from the apex and obtained a 2 mm thick ring. We placed the ring in a 10% buffered formaldehyde solution, which remained there for 24 h. We inserted the sections in paraffin and subsequently cut them with a thickness of 4 μm and stained them with hematoxylin and eosin (HE). We used HE-stained histological sections to analyze tissue integrity, the presence of inflammatory infiltrates, and the myocyte cross-sectional area using the ImageJ program (Wayne Rasband—National Institutes of Health, Bethesda, MD, USA). 

### 2.7. Myocardial Energy Metabolism

We homogenized samples of left ventricular tissue (100 mg) in a sodium phosphate buffer (pH 7.0, 0.1 M) and centrifuged them at 12,000 rpm for 30 min at −4 °C. We used supernatants to determine lactate dehydrogenase (LDH), phosphofructokinase (PFK), pyruvate dehydrogenase (PD), citrate synthase (CS), β-hydroxyacyl-CoA dehydrogenase (BHACD), Complex I (NADH: ubiquinone oxidoreductase), Complex II (succinate dehydrogenase), and ATP synthase, as previously described [5,33,34,35]. We determined all enzymatic activity in the myocardium by spectrophotometry, with readings performed on a mQuant microplate reader (BioTek Instruments, Winooski, VT, USA). We obtained all reagents from Sigma-Aldrich (Saint Louis, MO, EUA).

### 2.8. Oxidative Stress

We used left ventricular tissue samples (50 mg) to measure lipid hydroperoxide, as a marker of oxidative damage, glutathione peroxidase (E.C.1.11.1.9.), and superoxide dismutase (E.C.1.15.1.1.) to measure antioxidant enzymatic activity (EC.1.11.1.6.). In brief, we homogenized sample tissue in sodium phosphate buffer pH = 7.4 (0.01 M) and then centrifuged it at 12,000 rpm for 30 min at −4 °C. We used the supernatants to determine lipid hydroperoxide concentration by spectrophotometry [36]. Additionally, the same supernatants contained the antioxidant enzymes by spectrophotometry described previously [37,38]. 

### 2.9. Statistical Analyses

Data are expressed as mean ± SD when presented through normal distribution. We normalized variables with non-normal distribution by the mathematical transformation to obtain normal distribution, an essential condition to performing a two-way analysis of variance (ANOVA). We also presented the non-normalized data as mean ± SD. We performed a statistical analysis by a two-way ANOVA. Only when we found an interaction between two factors (Dox and doxycycline) did we perform comparisons between groups by Holm–Sidak (C vs. D, C vs. IM, IM vs. DIM, and D vs. DIM). When there was no interaction between the factors, we presented marginal data (the Dox effect alone or the doxycycline effect alone, without comparisons between groups). The significance level adopted was 5% for all analyses.

## 3. Results 

### 3.1. Body Weight and Fluid and Chow Intake

**Figure 1:**
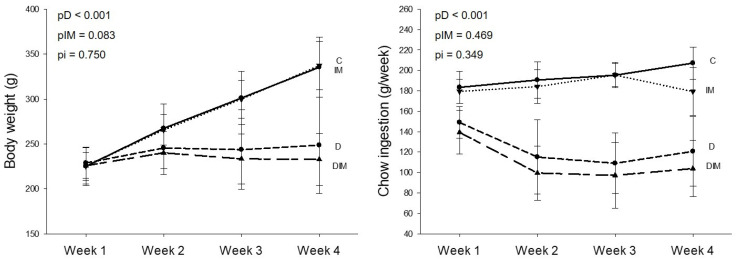
Body weight and chow intake. C, control group (*n* = 18); D, group treated with Dox (*n* = 20); IM, group treated with doxycycline (*n* = 19); DIM, group treated with Dox + doxycycline (*n* = 21). Initial, body weight immediately before the beginning of the experiment. We measured first food intake 1 week after drug administration. *p*-value, comparison between groups in week 4 by two-way ANOVA; pD, *p*-value for Dox effect; pIM, *p*-value for doxycycline effect; pi, *p*-value for the interaction between the factors Dox and doxycycline.

At the initial phase, the animals had a similar body weight. After the first week, the animals that received Dox showed weight loss and lower feed intake, extending until the end of the experiment, as seen in Figure 1.

### 3.2. Echocardiogram

The Dox treatment increased the left ventricular (LV) systolic diameter, the left atrial (LA) diastolic diameter, the LA diastolic diameter/aorta ratio, the LV mass index, and the isovolumetric relaxation time. Moreover, Dox treatment promoted a decrease in E-wave and LV fractional shortening. During the tissue Doppler, Dox treatment decreased E’ and S’ and increased the E’/E ratio. When Dox was administered in association with the doxycycline, we observed a decrease in the LA diastolic diameter and the LA diastolic diameter/aorta ratio and an increase in the E’ wave, which characterized improvement in diastolic function. Additionally, the DIM group exhibited increases in LV fractional shortening and S’ when compared with the D group, which characterized improvement in systolic function (Table 1).

### 3.3. Isolated Heart Study

There was no difference in the initial balloon volume; the maximum systolic pressure; the maximum left ventricular pressure development rate (+dP/dt), which is related to systolic function; or maximum left ventricular pressure decrease rate (−dP/dt), which is related to the diastolic function between the groups, as displayed in Table 2.

### 3.4. Collagen Type I and TIMP-4 Protein Expression

**Figure 2:**
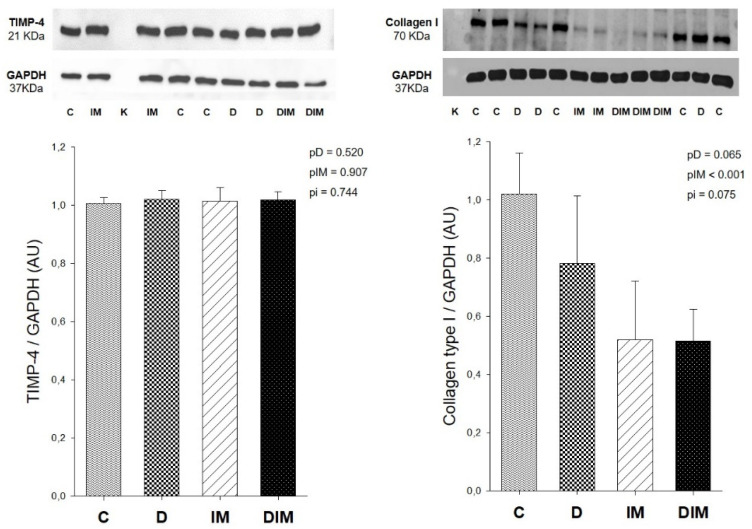
Collagen I and TIMP-4 protein expression. C, control group (*n* = 9); D, group treated with Dox (*n* = 8); IM, group treated with doxycycline (*n* = 9); DIM, group treated with Dox + doxycycline (*n* = 8); K, kaleidoscope molecular weight standard; UA, arbitrary units; *p*-value, two-way ANOVA; pD, *p*-value for Dox effect; pIM, *p*-value for doxycycline effect; pi, *p*-value for the interaction between the factors Dox and doxycycline.

TIMP-4 expression was similar between the groups. Dox treatment did not affect collagen type I expression; however, the animals that received the MMP inhibitor did show a decrease in collagen type I expression (Figure 2).

### 3.5. MMP-2 Activity

**Figure 3:**
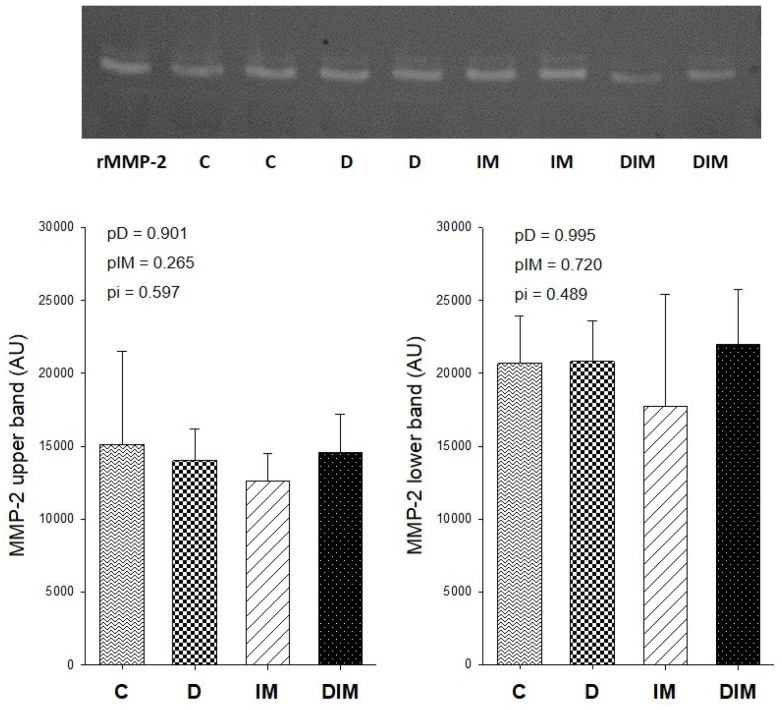
MMP-2 activity. rMMP-2, MMP-2 recombinant used as a positive control; C, control group (*n* = 11); D, group treated with Dox (*n* = 8); IM, group treated with doxycycline (*n* = 8); DIM, group treated with Dox + doxycycline (*n* = 9); UA, arbitrary units; *p*-value, two-way ANOVA; pD, *p*-value for Dox effect; pIM, *p*-value for doxycycline effect; pi, *p*-value for the interaction between the factors Dox and doxycycline.

There was no difference in the MMP-2 activity among groups assessed through zymography. Additionally, we did not observe the effect of doxycycline on MMP activity, as demonstrated in Figure 3.

### 3.6. Histology

**Figure 4:**
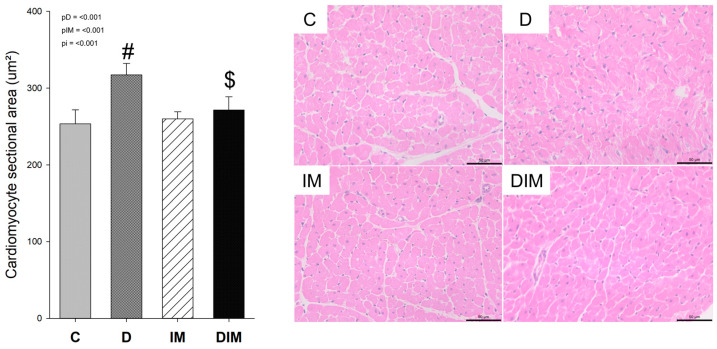
Histology and Cardiomyocyte Sectional Area. C, control group (*n* = 8); D, group treated with Dox (*n* = 8); IM, group treated with doxycycline (*n* = 8); DIM, group treated with Dox + doxycycline (*n* = 8); *p*-value, two-way ANOVA; pD, *p*-value for Dox effect; pIM, *p*-value for doxycycline effect; pi, *p*-value for the interaction between the factors Dox and doxycycline; #, different from C group; $, different from D group.

We did not observe the presence of inflammatory infiltrate or changes in the myocardial structural organization in any group. However, the D group had a greater cardiomyocyte sectional area when compared with the C group, and the DIM group had a smaller sectional area when compared with the D group, which may suggest that the doxycycline was able to attenuate the LV cardiomyocyte hypertrophy, as seen in Figure 4.

### 3.7. Myocardial Energy Metabolism

**Figure 5:**
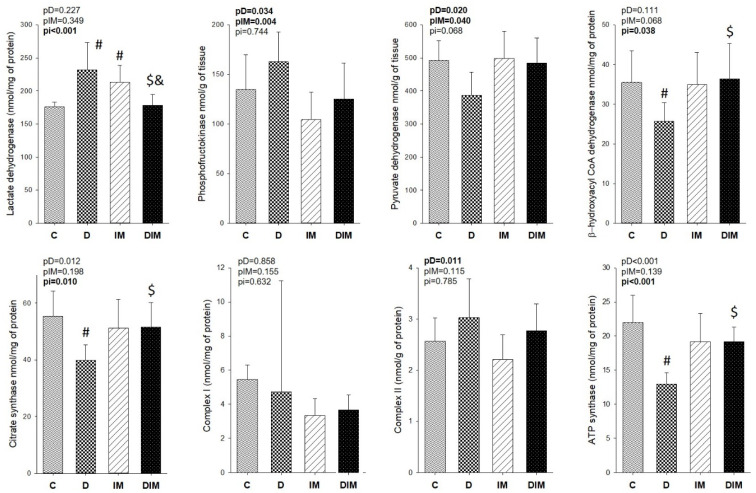
Energy myocardial metabolism. C, control group (*n* = 11); D, group treated with Dox (*n* = 9); IM, group treated with doxycycline (*n* = 9); DIM, group treated with Dox + doxycycline (*n* = 10). *p*-values are from a two-way analysis of variance; pD, *p*-value for Dox effect; pIM, *p*-value for doxycycline effect, pi, *p*-value for the interaction between the factors Dox and doxycycline; #, different from C group; $, different from D group; &, different from IM group.

Dox treatment promotes increases in the activity of complex II of the mitochondrial respiratory chain, lactate dehydrogenase, and phosphofructokinase enzymes, of which these last two are related to cellular glucose metabolism. Additionally, we observed a decrease in beta-hydroxyacyl Co-A dehydrogenase, pyruvate dehydrogenase, citrate synthase, and ATP synthase activity in animals treated with Dox (*p*-value for the Dox factor < 0.005). Doxycycline was able to improve lactate dehydrogenase, citrate synthase, beta-hydroxyacyl Co-A dehydrogenase, and ATP synthase activity, partially attenuating the alterations caused by Dox in cell metabolism (see Figure 5).

### 3.8. Oxidative Stress

**Figure 6:**
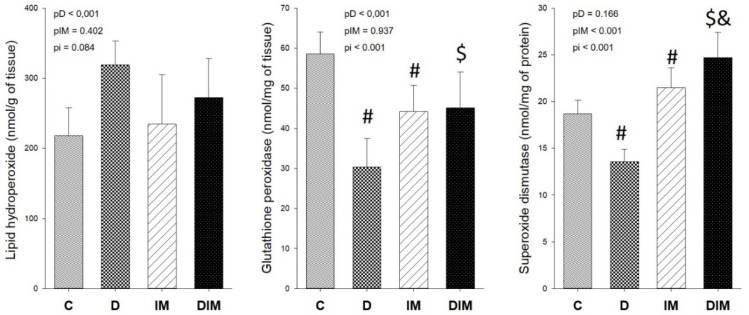
Oxidative stress. C, control group (*n* = 11); D, group treated with Dox (*n* = 9); IM, group treated with doxycycline (*n* = 9); DIM, group treated with Dox + doxycycline (*n* = 10). *p*-value, two-way ANOVA; pD, *p*-value for Dox effect; pIM, *p*-value for doxycycline effect; pi, *p*-value for the interaction between the factor Dox and doxycycline; #, different from C group; $, different from D group; &, different from IM group.

Rats treated with Dox exhibited higher levels of myocardial lipid hydroperoxide, accompanied by a decrease in the activity of the antioxidant enzymes superoxide dismutase and glutathione peroxidase. The combined administration of Dox and doxycycline did not reduce the oxidative damage given that we did not observe differences between the D group and the DIM group, but doxycycline increased the activity of superoxide dismutase and glutathione peroxidase, as seen in Figure 6.

## 4. Discussion

Increased oxidative stress has been implicated in the MMPs’ activation and also in the onset of cardiotoxicity induced by Dox [11,13,31]. We demonstrated here that the treatment with Dox promoted myocardial structural changes, systolic and diastolic dysfunction in vivo, increased oxidative stress, and a shift in myocardial metabolism. On the other hand, administration of doxycycline attenuated the structural changes and cardiac dysfunction apart from improving, at least partially, the changes in energy metabolism caused by Dox.

Currently, Dox is one of the most studied chemotherapy drugs due to its efficacy in treating a wide range of cancers. However, Dox administration is related to a variety of side effects in many tissues and organs, such as the liver, the kidneys, the skeletal muscles, and the heart, its preferential target of toxicity [39,40]. Additionally, Dox could cause gastrointestinal side effects such as inappetence, nausea, vomiting, and mucositis, commonly seen in humans [41]. In this study, as previously shown in other studies [5,31], Dox treatment promoted a decrease in food intake, followed by a decrease in body weight, which could be attributed to its gastrointestinal toxicity.

Doxycycline is a tetracycline antibiotic widely used to treat many different bacterial infections. Additionally, doxycycline is currently the only substance approved by the FDA as an MMP inhibitor for the treatment of periodontitis [42] and rosacea [43] at sub-antimicrobial doses. However, due to its wide range of nonantibiotic properties and relative safety profile, extensive pre-clinical and clinical studies have been investigating doxycycline therapeutic approaches, with promising results. Reports in several medical settings have shown ameliorating the severity of diabetic retinopathy [44] and proliferative vitreoretinopathy [45], inhibiting the proliferation and proapoptotic effect in human cancer cells [46,47,48,49], positive effects on osteoarthritis and posttraumatic arthritis progression [50], and reduction of cardiac dysfunction in different models of heart damage [22,51,52].

Previous studies have also observed that doxycycline was beneficial in the management of patients after ST-elevation myocardial infarction (STEMI) in reducing LV remodeling [53]. However, its effects in the clinical setting of cardiotoxicity induced by Dox are still unknown.

When we analyzed the cardiac function in vivo by echocardiogram, we observed cardiac chamber dilatation accompanied by systolic and diastolic dysfunction in rats that received Dox. Doxycycline was able to attenuate these changes. This result was also demonstrated recently by Chan et al. [23], where MMP inhibition with doxycycline ameliorated systolic and diastolic dysfunction by reducing the loss in the left ventricular ejection fraction, fractional shortening, and E’/A’ in mice treated with Dox for 4 weeks.

The functional improvement evidenced by echocardiography analyses was not reproduced when we evaluated ex vivo cardiac function by the isolated heart study technique. However, it is noteworthy that results obtained in this technique are under no influence of intrathoracic pressure, neuroendocrine regulation, or inotropic effects. Hence, we suggest that the ventricular dysfunction observed in the model of Dox-induced cardiotoxicity depends on factors other than the specific properties of the myocardium and its contractile capacity.

Following the morphological changes on the echocardiogram, we identified cardiomyocyte hypertrophy only in the D group, which may occur as an adaptive response to the functional overload exerted on the heart [54]. In the cardiac remodeling process, there is an increase in protein synthesis as a result of pressure and/or volume overload, leading to interstitial fibrosis and modification of the interstitial matrix composition [55].

The cardiac extracellular matrix is composed primarily of collagen type I, accounting for approximately 85% to 90% of the collagenous matrix that provides mechanical support and regulates several cellular functions of cardiovascular tissue [56]. Although we did not observe differences in collagen protein expression in cardiac tissue exposed to Dox, we identified a decrease in collagen type I protein expression among animals that received the doxycycline, as shown previously [57]. Given that collagen type I content is related to myocardial stiffness, the lowest collagen type I content could be contributed to the attenuation of LV dysfunction.

Collagens and other extracellular matrix proteins dynamics are regulated by several factors but mostly by MMP activity. In situations of cardiac injury, a large increase in the proteolytic activity of MMPs occurs, promoting changes in the cardiac matrix composition [12]. The tissue inhibitor of metalloproteinases 4 (TIMP-4) expressed in the heart binds to MMP-2 and -9, and thus, it regulates its activity. Additionally, TIMPs seem to be involved in other tissue processes, such as cellular proliferation and apoptosis [58,59].

Contrary to our expectations, we did not observe differences in MMP-2 activity or TIMP-4 protein expression in cardiac tissue, which could explain the decrease in collagen I expression among animals that received doxycycline. However, collagen synthesis and degradation can be regulated by other cellular types, such as fibroblasts, and other pathways, such as MMP-9, TIMP-2, the renin-angiotensin system, and transforming growth factor-β (TGF-β), which were not evaluated in this study.

TIMP-2 has the specific ability to stimulate collagen synthesis, and MMP-9 seems to be more activated in heart failure with reduced ejection fraction and dilated cardiomyopathies than MMP-2 [60]. Transforming growth factor-β (TGF-β) is one of the most important profibrotic stimuli in several tissues. It also regulates cell proliferation, apoptosis, differentiation, autophagy, and the immune response [61]. In the experimental model of renal disease induced by cisplatin, doxycycline decreased gene expression for TGF-β [62]; it may be the pathway related to the decrease of collagen type I expression in our study.

Moreover, it may be that MMP-2 activation occurs earlier and more acutely to allow the rearrangement of myofibrils and supporting tissue that accompanies the process of cardiac remodeling and dilation. Because we evaluated the animals in a chronic model, the balance between MMP activation and extracellular matrix formation and deposition may already have been balancing.

Cardiac injury leads to a shift in heart energy metabolism; the myocardium starts to use mainly glucose to generate ATP because it is less costly for the tissue than fatty acids. Concerning this, Dox treatment increased the activity of enzymes related to glucose metabolism (phosphofructokinase and lactate dehydrogenase) and decreased the activity of enzymes related to lipid metabolism (β-hydroxyacyl Co-A dehydrogenase) as well as citrate synthase activity (the first enzyme of the Krebs cycle) and ATP synthase of the mitochondrial phosphorylation, as demonstrated in our previous studies [5,31]. The administration of doxycycline attenuated these metabolic alterations, bringing the myocardial metabolism closer to the physiological situation; we observed an improvement in the activity of lactate dehydrogenase, β-hydroxyacyl Co-A dehydrogenase, citrate synthase, and ATP synthase.

The exact mechanism behind this improvement is not completely understood. It may have been mediated by the immunomodulation promoted by doxycycline [62,63,64]. Additionally, doxycycline treatment decreased the mitochondrial fragmentation induced by oxidative stress and improved the protein expression of molecules related to mitochondrial dynamics in the model of heart failure secondary to isoproterenol administration [65].

Energy metabolism changes and the impairment of mitochondrial oxidative phosphorylation can lead to increases in the generation of reactive oxygen species and, consequently, rising oxidative stress. Indeed, Dox treatment promoted greater levels of lipid hydroperoxide, considered a marker of oxidative damage to the lipids of the cellular membrane. Despite the supposed improvement in mitochondrial function, doxycycline did not decrease the cardiac lipid hydroperoxide concentration; rather, it increased superoxide dismutase (SOD) and glutathione peroxidase (GPx) activity.

It was already reported that doxycycline could directly increase SOD gene expression [62] and the enzymatic activity of SOD and GPx in heart tissue [29]. Moreover, doxycycline was able to counteracts doxorubicin-imposed oxidative stress in a model of acute cardiotoxicity induced by Dox, exhibiting cytoprotective properties by both decreasing reactive oxygen species generation and elevating SOD and GPx activities [29]. Similarly, an increase in the antioxidant enzymes and decrease in oxygen species production was also observed after doxycycline treatment in different experimental models [26,66].

Additionally, this antioxidant property could be related to a decrease in the activation of Nrf-2 (a redox-sensitive transcription factor), the modulation of NF-kB activity (related to the antioxidant enzymes and transcription of inflammatory cytokines), and the well-established doxycycline’s effect in reducing the levels of a range of pro-inflammatory cytokines such as IL-1β, IL-6, IL-8, and TNF-α [27,67]. Unfortunately, we did not evaluate the inflammatory status in the present study.

Although we did not completely elucidate the specific mechanism involved in beneficial effect of doxycycline in Dox-induced cardiotoxicity, it seems to be related to the changes in myocardial energy metabolism and oxidative stress.

## 5. Conclusions

The chronic administration of Dox caused an increase in oxidative stress and promoted changes in myocardial energy metabolism, accompanied by structural and functional cardiac alterations characterizing cardiotoxicity. Even though doxycycline did not modify MMP-2 activity or TIMP-4 expression in this chronic model, its administration attenuated the Dox-induced cardiotoxicity, at least in part, for the changes in myocardial energy metabolism and the decrease in myocardial collagen type I content. These unprecedented results suggest a promising therapeutic property of doxycycline’s protection against cardiotoxicity caused by Dox and demonstrate its potential benefit in targeting multiple pathways involved in cardiac remodeling, which lends us to an interesting perspective in the future research.

## Figures and Tables

**Table 1:** Morphological variables and cardiac function evaluated by echocardiogram.

Variables	C (*n* = 18)	D (*n* = 19)	IM (*n* = 17)	DIM (*n* = 20)	pD	pIM	pi
HR (bpm)	302 ± 62.60	261 ± 56.80	302 ± 52.50	270 ± 83.8	0.020	0.762	0.764
LVSD (mm)	2.65 ± 0.45	3.51 ± 0.64 ^#^	2.82 ± 0.18	2.90 ± 0.49 ^$^	<0.001	0.050	<0.001
LVDD (mm)	6.60 ± 0.48	6.49 ± 0.76	6.80 ± 0.53	6.40 + 0.43	0.054	0.701	0.278
LADD (mm)	4.50 ± 0.18	4.96 ± 0.46 ^#^	4.62 ± 0.16	4.38 ± 0.30 ^$&^	0.006	0.056	0.006
Aorta (mm)	3.58 ± 0.16	3.44 ± 0.16	3.62 ± 0.14	3.47 ± 0.18	<0.001	0.434	0.821
LADD/aorta	1.26 ± 0.06	1.44 ± 0.11 ^#^	1.28 ± 0.04	1.26 ± 0.08 ^$^	<0.001	<0.001	<0.001
LVMI (g/kg)	1860 ± 275	2131 ± 345	1956 ± 198	2391 ± 230	<0.001	0.006	0.196
LVFS	0.60 ± 0.05	0.46 ± 0.07 ^#^	0.58 ± 0.03	0.54 ± 0.06 ^$^	<0.001	0.009	<0.001
E wave (cm/s)	80.2 ± 16.00	66.4 ± 11.60	79.9 ± 8.99	75.3 ± 13.00	0.003	0.148	0.120
A wave (cm/s)	52.5 ± 16.30	47.0 ± 18.20	54.3 ± 12.80	47.7 ± 13.10	0.218	0.488	0.155
IVRT (ms)	23.8 ± 2.62	35.6 ± 6.42	22.7 ± 1.36	29.3 ± 6.42	0.006	0.294	0.160
E’m (cm/s)	5.75 ± 0.63	3.84 ± 1.23 ^#^	5.35 ± 0.80	4.81 ± 1.78 ^$^	<0.001	0.320	0.019
A’m (cm/s)	3.72 ± 0.33	4.07 ± 0.87	3.31 ± 0.72	4.33 ± 0.69 ^&^	<0.001	0.638	0.040
E/E’m	13.9 ± 1.84	18.4 ± 4.92	15.1 ± 1.64	17.0 ± 4.07	<0.001	0.872	0.109
S’m (cm/s)	5.74 ± 0.28	4.22 ± 0.79 ^#^	5.58 ± 0.35	4.79 ± 0.86 ^$&^	0.006	0.160	0.006

C, control group; D, group treated with Dox; IM, group treated with doxycycline; DIM, group treated with Dox + doxycycline; LVSD, left ventricular (LV) systolic diameter; LVDD, LV diastolic diameter; LADD, left atrial diastolic diameter; LVMI, LV mass index; LVFS, LV fractional shortening; E, peak velocity of early ventricular filling; A, peak velocity of transmitral flow during atrial contraction; IVRT, isovolumetric relaxation time; E’m, mean of displacement of the mitral ring in the lateral and septal walls during initial diastole in tissue Doppler; A’m, mean of displacement of the mitral ring in the lateral and septal wall during late diastole in tissue Doppler; S’m, mean of displacement of the mitral ring in the lateral and septal wall during systole in tissue Doppler. *p*-value, two-way ANOVA; pD, *p*-value for Dox effect; pIM, *p*-value for doxycycline effect; pi, *p*-value for the interaction between the factors Dox and doxycycline. ^#^, different from C group; ^$^, different from D group; ^&^, different from IM group.

**Table 2:** Left ventricular function evaluated by isolated heart study.

Variables	C (*n* = 8)	D (*n* = 5)	IM (*n* = 6)	DIM (*n* = 8)	pD	pIM	pi
V_0_	75.0 ± 22.2	56.0 ± 16.4	67.5 ± 18.9	61.3 ± 24.6	0.147	0.895	0.456
+dP/dt	5219 ± 743.3	4100 ± 1413	3833 ± 1211	3641 ± 1407	0.177	0.062	0.335
−dP/dt	3891 ± 362.5	3175 ± 1134	3063 ± 1078	2719 ± 903.2	0.136	0.074	0.593
SP	201 ± 24.0	174.5 ± 43.4	164 ± 39.6	156 ± 37.0	0.230	0.071	0.540

C, control group; D, group treated with Dox; IM, group treated with doxycycline; DIM, group treated with Dox + doxycycline; V_0_, initial balloon volume; +dP/dt, maximum left ventricular pressure development rate; −dP/dt, maximum left ventricular pressure decrease rate; SP, maximum systolic pressure data are expressed as mean ± SD. *p*-values are from two-way analysis of variance; pD, *p*-value for Dox effect, pIM, *p*-value for doxycycline effect; pi, *p*-value for the interaction between the factors Dox and doxycycline.

## Data Availability

Data are contained within the article. The data presented in this study are available with the corresponding author.
